# The Marine-Origin Exopolysaccharide-Producing Bacteria *Micrococcus Antarcticus* HZ Inhibits Pb Uptake in Pakchoi (*Brassica chinensis* L.) and Affects Rhizosphere Microbial Communities

**DOI:** 10.3390/microorganisms12102002

**Published:** 2024-10-01

**Authors:** Nan Liu, Gangrui Zhang, Longyu Fang, Rui Geng, Shengbo Shi, Jinghua Li, Wei Wang, Mingchun Lin, Junfeng Chen, Yanru Si, Zeyun Zhou, Baoli Shan, Maoyu Men, Qisheng Fan, Chengze Wang, Chaoqun Zhang, Lujiang Hao

**Affiliations:** 1 School of Bioengineering, Qilu University of Technology (Shandong Academy of Sciences), Jinan 250353, China; 10431221234@stu.qlu.edu.cn (N.L.);; 2 Jiangsu Key Laboratory for Microbes and Functional Genomics, Engineering and Technology Research Center for Microbiology, College of Life Sciences, Nanjing Normal University, Nanjing 210000, China; 3 Shandong Pacific Environmental Protection Co., Ltd., Jinan, China

**Keywords:** marine bacteria, exopolysaccharides (EPSs), bioremediation, Pb-immobilizing, heavy-metal-contaminated soil, bacterial community

## Abstract

Exopolysaccharides (EPSs) produced by microorganisms play an important role in biotolerance and reducing heavy metal (HM) contamination by limiting the migration of HMs into plants. However, research on the application of EPS-producing marine bacteria for soil heavy metal remediation remains limited, particularly regarding their mechanisms of HM immobilization in soil and impact on plant growth. In this study, the EPS-producing marine bacterium *Micrococcus antarcticus* HZ was investigated for its ability to immobilize Pb and produce EPSs in soil filtrate. The effects on the growth quality and biomass of pakchoi (*Brassica chinensis* L.), as well as bacterial communities in inter-root soil contaminated with Pb, were also investigated. The results indicated that HZ could reduce the Pb concentration in the soil filtrate, achieving a removal rate of 43.25–63.5%. The EPS content and pH levels increased in the presence of Pb. Pot experiments showed that adding HZ significantly increased the biomass of pakchoi (9.45–14.69%), vitamin C (Vc) (9.69–12.92%), and soluble protein content (22.58–49.7%). HZ reduced the Pb content in the roots (17.52–47.48%) and leaves (edible tissues) (43.82–52.83%) of pakchoi. HZ increased soil enzyme activities (alkaline phosphatase, dehydrogenase, and urease), and the contents of ammonium nitrogen and nitrate nitrogen. Additionally, HZ also increased the relative abundance of beneficial bacteria (e.g., Proteobacteria, Cyanobacteria, and Chlorobacteria) in the inter-root soil, which have prophylactic and heavy-metal fixation functions. In summary, HZ reduces effective Pb content in edible tissues, roots, and inter-root soil by regulating inter-root soil microbial community structure, increasing soil pH, nitrogen content, and soil enzyme activity, and altering dominant phylum abundance.

## 1. Introduction

Heavy metal pollution in soil has numerous origins and widespread distribution, with lead (Pb) and cadmium (Cd) being among the most serious and harmful contaminants [1,2,3]. Heavy metals in soil enter the human body through the food chain and jeopardize human health. Excessive intake of lead would increase the incidence of dental caries and could change the nervous system, leading to loss of nerve function, resulting in brain damage and anemia in children [4]. Therefore, it is crucial to investigate strategies for preventing and controlling soil contamination with heavy metals. Various methods have been used to eliminate heavy metal pollution in soil. Physicochemical remediation methods have certain limitations, although they can reduce heavy metal pollution in soil to a certain extent. Because most of these techniques are invasive, they can disrupt the equilibrium of the ecosystem and impact the environment [5,6]. Therefore, compared with chemical and physical remediation, microbial remediation techniques are low-cost and do not harm the soil, and have become a common remediation method [7,8,9].

In recent years, researchers have increasingly focused on using soil-immobilizing bacteria, a novel type of soil-immobilizing agent, to reduce the effectiveness of metals in the soil and their uptake by plants [10,11,12]. The mechanism of heavy metal adsorption by bacteria is complex [13,14,15]. For instance, bacteria can secrete extracellular substances, such as polysaccharides, proteins, and lipids, to precipitate heavy metals [16,17,18]. HMs are immobilized on the bacterial cell surface by binding to the cell surface structures. Additionally, bacteria can internally detoxify heavy metals by facilitating their exocytosis, obstructing the influx of heavy metals, converting heavy metals, and compartmentalizing them within the cell. These processes are interconnected rather than isolated [19].

When faced with heavy metal stress or nutritional imbalance, bacteria are stimulated to synthesize and secrete EPSs [20,21,22]. EPS-producing bacteria can enhance the tolerance of host plants, thereby improving plant growth [17,23,24,25]. Additionally, Jiang et al. verified that the cyanobacterium *Nostoc spheroid* can produce large amounts of extracellular polysaccharides and effectively adsorb toxic metals, making it suitable for industrial wastewater treatment [26]. MAL Huët et al. found that *Enterococcisp.* MC1 could remove Pb and Cd from water bodies, while *Bacillus acidiproducens* SM2 was the most effective in removing Chromium (Cr) [27]. Two EPS-producing bacteria, *Bacillus gibsonii* (PM11) and *Bacillus xiamenensis* (PM14), were able to increase flax plant growth and nutrient utilization, minimizing metal-induced stress conditions [24].

The utilization of polysaccharide-producing bacteria for heavy metal remediation has garnered significant interest among researchers [21]. However, research on the application of polysaccharide-capable marine bacteria for soil metal contamination remediation is lacking. Marine bacteria, which live in extreme environments with low temperatures and high pressures, possess a superior capability to generate unique bioactive compounds, such as EPSs, compared to other bacteria [25,28,29,30]. EPSs produced by marine bacteria are particularly interesting because they typically contain higher levels of glyoxalate, which makes them more anionic. This anionic nature endows EPSs with excellent chelating ability, especially for metal ions. In addition, marine bacteria exhibit remarkable osmotolerance, which allows them to efficiently synthesize EPSs in environments with high sugar concentrations. Hence, the utilization of polysaccharide-producing marine bacteria for soil metal pollution remediation holds significant importance. The uptake and accumulation of heavy metals by leafy vegetables might represent a primary route for human exposure to these contaminants. Among leafy vegetables, pakchoi is extensively cultivated in China, accounting for 19% of the total output of leafy vegetables [31].

To investigate the impact and mechanism of in situ remediation of soil heavy metals using polysaccharide-producing marine bacteria, we used a strain of the marine bacterium *Micrococcus antarcticus* HZ, known for its high production of extracellular polysaccharides, and Chinese cabbage as a test plant. We studied how strain HZ inhibited Pb uptake by Chinese cabbage. In this study, we assessed the adsorption effects of strain HZ on Pb in soil filtrate and its ability to produce EPS production, and examined the effects of strain HZ on the growth quality and biomass of pakchoi, along with the soil physicochemical properties, soil enzyme activities, soil nitrogen content, and inter-root soil microbial community in Pb-contaminated soil. This study enhances our understanding of the involvement of EPS-producing marine bacteria in soil microbial communities and Pb immobilization. It also helps reveal the mechanism behind soil heavy metal immobilization by EPS-producing bacteria and their impacts on soil microbes, establishing a scientific foundation for the advancement of safer and more efficient plant production techniques.

## 2. Materials and Methods

### 2.1. Bacteria, Soil, and Plants

A strain of marine bacteria isolated from abalone seedling collection plates in Rongcheng was identified as *Micrococcus antarcticus* (NCBI accession number: OP703333), named *Micrococcus antarcticus* HZ, and stored in our laboratory [32].

Soil samples were collected from the top 0–15 cm layer of uncontaminated soil at the Maize Research Institute of the Shandong Academy of Agricultural Sciences (N 36°44′, E 117°22′). The soil pH was 7.90 and the initial values of organic matter, total nitrogen, and total phosphorus content were 13.65, 1.41, and 2.42 g kg^−1^, respectively.

Pakchoi (*Brassica chinensis* L.) seeds were obtained from the Shandong Academy of Agricultural Sciences. Pakchoi is a leafy vegetable widely grown in China and is easily enriched in HMs.

### 2.2. Determination of Pb Concentration and EPS Yield in Soil Filtrate after Inoculation with Strain HZ

Soil samples (2.5 kg) were added to 10 L of deionized water, placed in a shaker, and shaken at 180 rpm for 48 h. The mixture was then centrifuged at 5000 rpm for 15 min. The supernatant was collected and filtered through a Millipore filter with a pore size of 0.45 um to remove bacteria, and the soil filtrate was obtained. Seed and fermentation media were configured, sterilized, and mixed with soil filtrate and sterile fermentation medium at a ratio of 4:1 to prepare a mixed fermentation broth, which was placed into 500 mL sterile conical flasks to simulate the soil environment and reserved for further use.

The activated strain, HZ, was inoculated into the seed medium and incubated for 8 h at 25 °C with shaking at 180 rpm. After washing thrice with sterile deionized water and resuspension in sterile saline, the resulting suspension was inoculated into a mixed fermentation broth containing varying concentrations of Pb^2+^ (0, 20, and 50 mg L^−1^). Three parallels were set in each group, with the uninoculated treatment serving as the control.

The cultures were incubated at 25 °C with shaking at 180 rpm for 96 h. Sampling points were set at 0, 24, 48, 72, and 96 h. Bacterial growth was detected by measuring optical density (OD_600_) at each time point. The pH value of the mixed fermentation broth was determined using a pH meter (PHS-3E, Shanghai Yidian Scientific Instrument Co., Ltd., Shanghai, China). Inductively coupled plasma mass spectrometry (ICP-MS) was used to measure Pb^2+^ concentrations, and the phenol–sulfuric acid method was employed to determine polysaccharide production.

### 2.3. Potting Experiments

The soil was dried in the shade and finely crushed with three concentrations (0, 25, and 50 mg L^−1^) established by thoroughly mixing PbCl_2_-2.5H_2_O with the soil. The soil was equilibrated under dark conditions for 45 days, maintaining a temperature of 25 °C and adding deionized water to keep the humidity at 70%. Each pot held 4.5 kg of processed soil and had a diameter of 28 cm and a height of 35 cm. The flotation method was used to select relatively full, similarly sized pakchoi seeds. The seeds were sterilized, and 30 intact seeds were evenly scattered on the surface of the soil in each pot and gently covered with fine soil. All treatments were placed in random rows in a greenhouse and rotated intermittently to maintain consistent growing conditions. After germination, 15 uniformly growing seedlings were retained in each pot and cultured continuously until the third true leaf of the pakchoi unfolded. The HZ strains were cultured in a seed medium for 8 h (25 °C, 180 rpm), then centrifuged, washed, and resuspended to a concentration of 1 ×10^8^ cells mL^−1^ using sterile water, with sterile water treatment as a control. Using a sterile syringe, 90 mL of the bacterial solution was injected into the root zone. Three parallels were made for each treatment and placed in a greenhouse to continue incubation for 45 days (temperature of 25 ± 3 °C, relative humidity of 70%, and an average photoperiod of 12 h/day).

### 2.4. Plant and Soil Sample Analysis

Soil tightly bound to the root system of pakchoi was brushed off with a soft-bristled brush and collected as inter-root soil. The rhizosphere soil samples were divided into three portions. The initial portion was air-dried and pulverized to analyze the soil sample’s pH and enzyme activity content. Subsequently, after drying and grinding, 0.05 MDTPA was used for extraction and the Pb content was measured using ICP-MS. The next section of the soil was preserved at 4 °C to analyze the NH_4_^+^-N and NO_3_^−^-N contents. The final segment of the soil was stored at −80 °C for total DNA extraction.

The collected chard was divided into two parts: the edible tissue and the root system. The cabbage leaves (edible tissues) and roots were cleaned and divided into two parts. The first portion was inactivated at 105 °C and dried at 65 °C until a constant weight was achieved. It was used to measure the dry weight and Pb content. The second part of the fresh leaves was used for analyzing vitamin C, soluble protein, and nitrite content following standard methods. The dried roots and edible parts of pakchoi were ground and digested in an acidic solution, and the bioavailable Pb content was assessed using ICP-MS.

### 2.5. Detection of Bacterial Strains Colonizing Inter-Root Soil

The soil suspension was serially diluted in potassium chloride solution, with three replicates set up. The diluted suspension was then spread on the prepared seed medium, and the bacteria were incubated in a constant-temperature incubator at 25 °C for 24 h. The HZ-like strains were counted, and to confirm that the colonies were HZ, approximately 10% of the isolates were randomly selected. The intergenic consensus sequences of Enterobacteriaceae were used as the primers for the identification of the rep-PCR method for their genetic fingerprints.

### 2.6. Analysis of Soil Flora

Bacterial genomic DNA was extracted from soil samples, and the quantity and quality of the extracted DNA were measured using a spectrophotometer and gel electrophoresis. The forward primer 338F (5′-ACTCCTACGGGGAGGAGCA-3′) and reverse primer 806R (5′-GGACTACHVGGTWTCAAT-3′) were used to amplify the 16SrRNA variable region. The PCR products were purified using the QiaQuick PCR purification kit, and high-throughput sequencing was performed using the Illumina HiSeq 2000 (Illumina Inc., San Diego, CA, USA).

### 2.7. Statistical Analysis

Treatment means were compared using one-way ANOVA and Tukey’s test (*p* < 0.05). Statistical analysis was conducted using the SPSS software (version 20.0; SPSS Inc., Chicago, IL, USA). The obtained sequence data were analyzed using Mothur software (1.47.0). The operational taxonomic units (OTUs) were classified using the UCLUST sequence tool. Community distribution diversity was estimated by calculating the Shannon, Simpson, Chao1, and ACE indices. Principal Coordinate Analysis (PCoA) of the microbial communities was performed on the distance matrix, which was used to generate two-dimensional graphical results. Taxa abundances at the phylum, order, family, and genus levels were statistically compared between samples and groups using Metastats.

## 3. Results

### 3.1. Determination of Concentrations of Water-Soluble Pb and EPS in Soil Filtrate after Inoculation with Strain HZ

**Figure 1:**
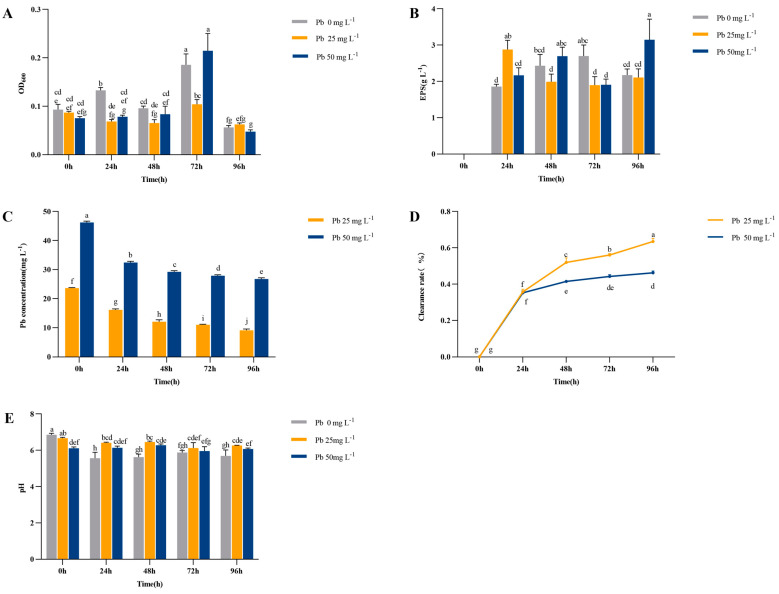
The cell number (indicated by OD600) (**A**), EPS (**B**), Pb (**C**), content clearance rate of Pb (**D**), and PH (**E**) in the culture solution at different Pb concentrations inoculated with HZ. Each bar represents the mean ± SD (n = 4). The same letter indicates that there were no significant differences between all treatments (*p* > 0.05).

The biomass of strain HZ usually increased with time in the treatment groups with different Pb contents, reaching peaks of 1.86, 1.04, and 2.15 at 72 h, respectively (Figure 1A). The findings showed that the growth lag phase of strain HZ was extended following exposure to Pb^2+^ stress induced by the application of Pb. The growth lag period of HZ was 48 h when the Pb concentration was 25 mg L^−1^ as well as 50 mg L^−1^. Notably, the growth rate of HZ at 25 mg L^−1^ of Pb remained consistently low across all time intervals, falling below that of the control group. The results of the EPS production indicated a positive correlation between the biomass of HZ and the amount of EPSs produced in the control group, both reaching peak values at 72 h (1.86 and 2.70 g L^−1^) (Figure 1B). Under low Pb concentration stress (25 mg L^−1^), the growth conditions and EPS production of HZ were relatively flat. However, at 50 mg L^−1^ Pb, the EPS production of HZ showed an upward trend, peaking at 96 h (3.15 g L^−1^). After inoculation with HZ, the soluble Pb content decreased significantly over time (Figure 1C), and the highest clearance of Pb by HZ at 96 h was observed under the 25 mg L^−1^ and 50 mg L^−1^ treatments, which were 63.5% and 43.25%, respectively (Figure 1D). Additionally, the addition of Pb led to a decrease in the pH of the soil filtrate (Figure 1E), whereas HZ was able to increase the pH of the soil filtrate.

### 3.2. Colonization of Soil by Strain HZ

The colonization of the inter-root soil by strain HZ was analyzed, and single colonies were identified by ERIC-PCR genomic fingerprinting. The experimental results showed that 45 days after inoculation of strain HZ, the number of viable bacteria ranged from 2.17 to 3.33 × 10^4^ cfu g^−1^ fresh soil. This indicates that HZ can colonize the soil successfully.

### 3.3. Effect of Strain HZ on Available Pb in Rhizosphere Soil

**Figure 2:**
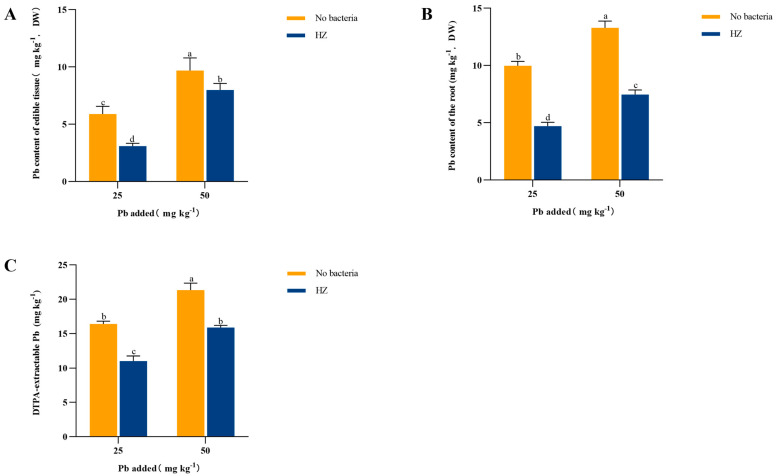
Effects of HZ on Pb content (**C**) of DTPA in edible tissue (**A**), roots (**B**), and rhizosphere soil of pakchoi. Each bar represents the mean ± SD (n = 4). The same letter indicates that there were no significant differences between all treatments (*p* > 0.05).

As the concentration of Pb in the soil increased, the Pb content in pakchoi also increased. However, inoculation with strain HZ notably decreased Pb levels in the edible tissues by 17.52–47.48% (Figure 2A) and root Pb content by 43.82–52.83% (Figure 2B). Furthermore, the introduction of HZ led to a significant decrease in the bioavailable Pb content in the soil when compared to the control group, The effective Pb content in the two treatment groups, Pb 25 mg kg^−1^ and Pb 50 mg kg^−1^, was reduced by 32.82% and 25.53%, respectively (Figure 2C).

### 3.4. Effect of Strain HZ on Cabbage Biomass and Edible Tissue Vitamin C, Soluble Protein, and Nitrite Content

**Figure 3:**
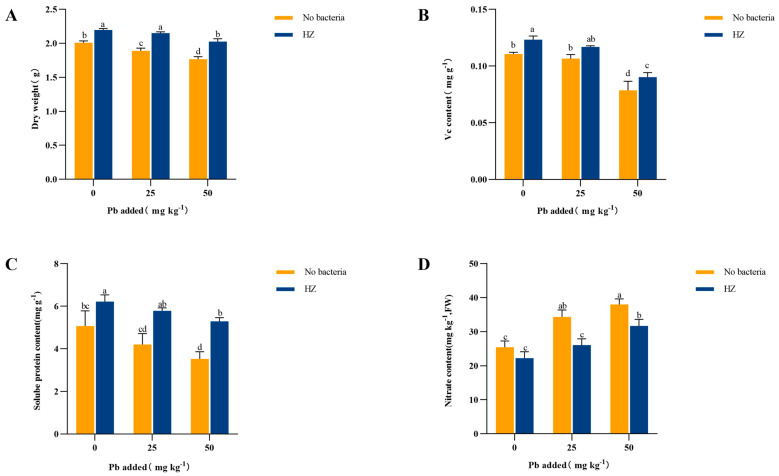
Effects of HZ on edible tissue biomass (**A**), Vc content (**B**), soluble protein content (**C**), and nitrite content (**D**) of pakchoi. Each bar represents the mean ± SD (n = 4). The same letter indicates that there were no significant differences between all treatments (*p* > 0.05).

Cabbage biomass gradually decreased with increasing Pb content (Figure 3A). In soils treated with low and high Pb concentrations, cabbage biomass decreased by 5.97% and 11.94%, respectively, compared to the control. The application of strain HZ increased cabbage biomass by 9.45% to 14.69%. Vitamin C (Vc) content and soluble protein content decreased with increasing Pb concentration, and HZ was able to increase the Vc content of cabbage edible tissues (Figure 3B) and soluble protein (Figure 3C). Specifically, at Pb concentrations of 0 mg kg^−1^, 25 mg kg^−1^, and 50 mg kg^−1^, Vc content increased by 11.44%, 9.69%, and 12.92%, respectively (Figure 3C). Soluble protein content increased by 22.58%, 37.55%, and 49.7%, respectively, compared to the control group. Nitrite content increased with increasing Pb concentration in the soil (Figure 3D), and the application of strain HZ significantly reduced the nitrite content by 12.5% to 24.14%.

### 3.5. Effect of Strain HZ on Soil Nitrogen Content and Soil Enzyme Activity

**Figure 4:**
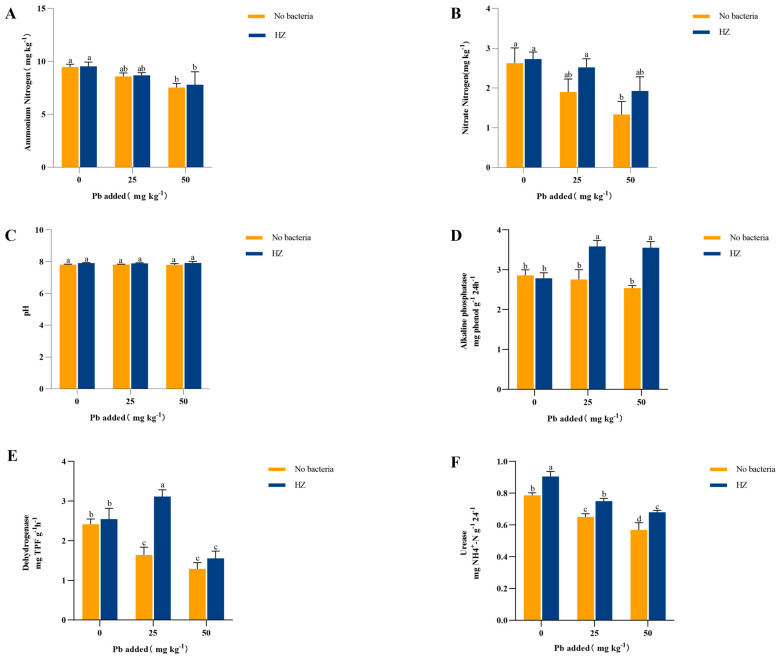
Effects of HZ on NH4+-N content (**A**), NO_3_-N content (**B**), pH (**C**), alkaline phosphatase content (**D**), dehydrogenase (**E**), and urease content (**F**) in rhizosphere soil of pakchoi. Each bar represents the mean ± SD (n = 4). The same letter indicates that there were no significant differences between all treatments (*p* > 0.05).

**Figure 5:**
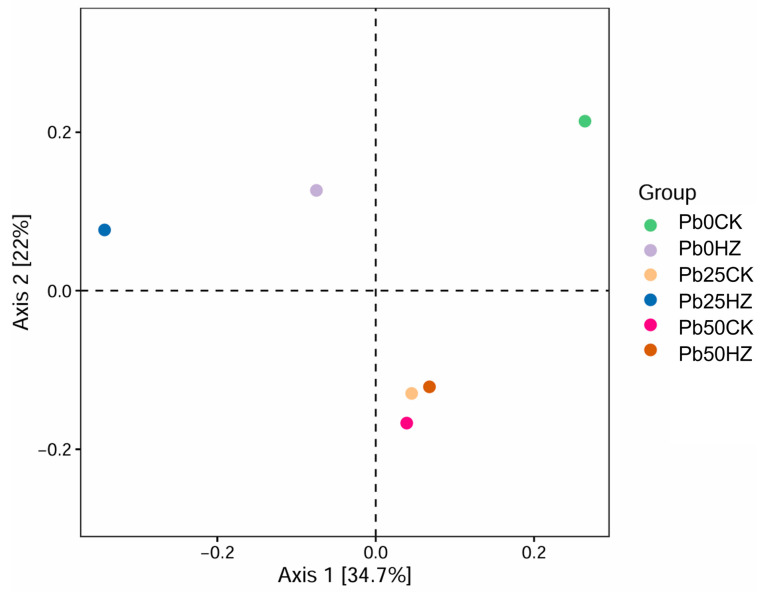
Analysis of principal coordinates (PCoA) of bacterial communities in soil samples under different treatments.

The effects of strain HZ on nitrogen content and pH in the soil around the roots are depicted in Figure 4. Irrespective of the presence of strain HZ, the NH_4_^+^ -N and NO_3_^−^ -N levels in the soil exhibited a declining pattern as the Pb concentration in the soil increased. However, the introduction of strain HZ resulted in an enhancement of the soil nitrogen content (Figure 4A,B). In comparison to the control group, inoculation with strain HZ resulted in a significant increase in NO_3_^−^ -N content by 14.89%, 32.75%, and 44.26%, respectively, with a greater increase observed at higher Pb concentrations. Additionally, soil pH increased after inoculation with strain HZ (Figure 4C). The effect of strain HZ on inter-root soil enzyme activities is shown in Figure 5. In the absence of strain HZ, there was no significant difference in soil alkaline phosphatase activity between Pb-contaminated and non-Pb-contaminated soils. However, the application of strain HZ to Pb-contaminated soil significantly increased the alkaline phosphatase content by 30.08% to 39.97%. Furthermore, dehydrogenase and urease activities in the inter-root soil decreased significantly with increasing soil Pb content in the absence of strain HZ. After inoculation with HZ, dehydrogenase activity increased by 5.35% to 89.68%, and urease activity increased by 15.06% to 19.88%, compared to the control group. Particularly noteworthy is the extremely significant increase in dehydrogenase activity (up to 89.68%) observed in the Pb 25 mg kg ^−1^ treatment group following the addition of HZ.

### 3.6. Bacterial Community Diversity and Structure

A total of 3,201,587 sequences were obtained from 16SrRNA high-throughput sequencing of inter-root soil bacterial samples of pakchoi in the potting experiment. As shown in Table 1, high-quality sequences were obtained which yielded an average of 72,196 high-quality sequences per sample.

The alpha diversity results are presented in Table 2. The good coverage of all samples reached more than 97%, indicating that the sequencing depth could reflect the microbial community structure more accurately. The effect of Pb application on the bacterial alpha diversity of inter-root soils was not significant, and in the inter-root soils at high content (Pb 50 mg kg^−1^), all indices of the applied strain HZ (Chao1, Shannon, Simpson, and Observed species) were significantly higher than those of the control, indicating that inoculation of HZ at the level of Pb 50 mg kg^−1^ increased the total number of species in the community, the diversity of the community, the diversity of the distribution of species within the community, and the evenness. The bacterial alpha diversity indices in the inter-root soil were lower than those in the control group at both Pb 0 mg kg^−1^ and Pb 25 mg kg^−1^, which indicated that inoculation of HZ at the levels of Pb 0 mg kg^−1^ and Pb 25 mg kg^−1^ decreased the inter-root microbial community structure of Brassica napus. This indicates that inoculation with HZ at the levels of Pb 0 mg kg^−1^ and Pb 25 mg kg^−1^ reduced the total number of community species, community diversity, diversity, and evenness of species distribution within the community.

To better observe the changes in the soil bacterial community composition between roots in the presence or absence of strain HZ, a PCoA analysis was performed, revealing that inoculation with strain HZ induced significant alterations in the bacterial community composition (Figure 5). The inter-root soil communities inoculated with strain HZ formed distinct clusters separate from those of the uninoculated controls, indicating a pronounced impact of strain HZ on the soil bacterial composition associated with pakchoi.

**Figure 6:**
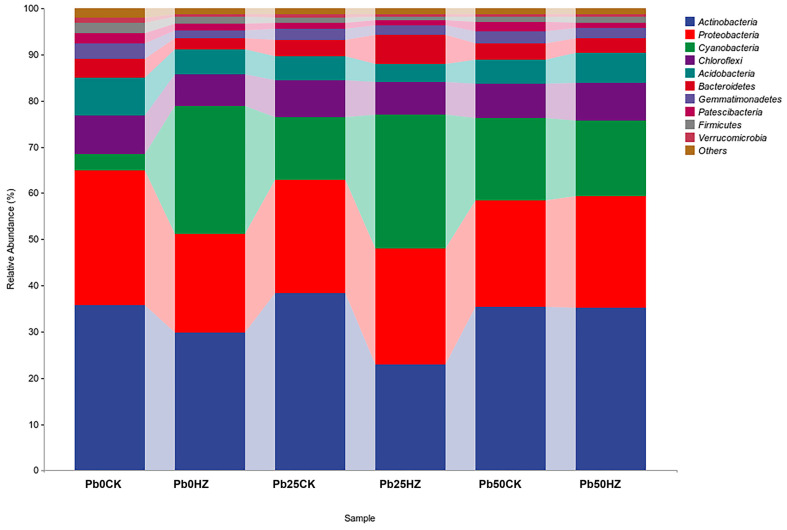
Relative abundance of bacterial communities in soil under different treatments.

A total of 72,196 high-quality sequences were obtained, representing 38 phyla (Figure 6). Across all soil samples, *Actinobacteria* (22.88–38.43%) and *Proteobacteria* (29.16–21.40%) were consistently dominant, followed by *Cyanobacteria* (3.52–29.11%), *Chloroflexi* (6.92–8.31%), and *Acidobacteria* (3.90–8.22%).

**Figure 7:**
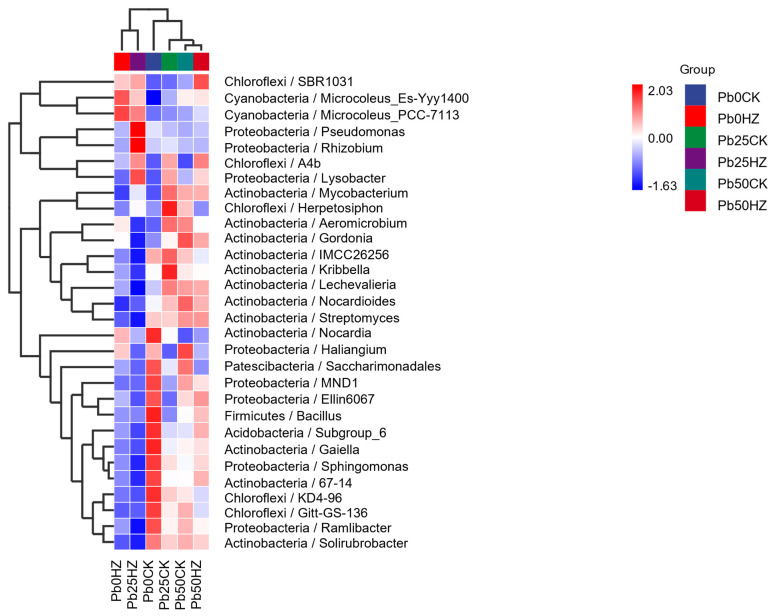
Heat maps of the top 30 genera of rhizosphere soil under different Pb pollution.

**Figure 8:**
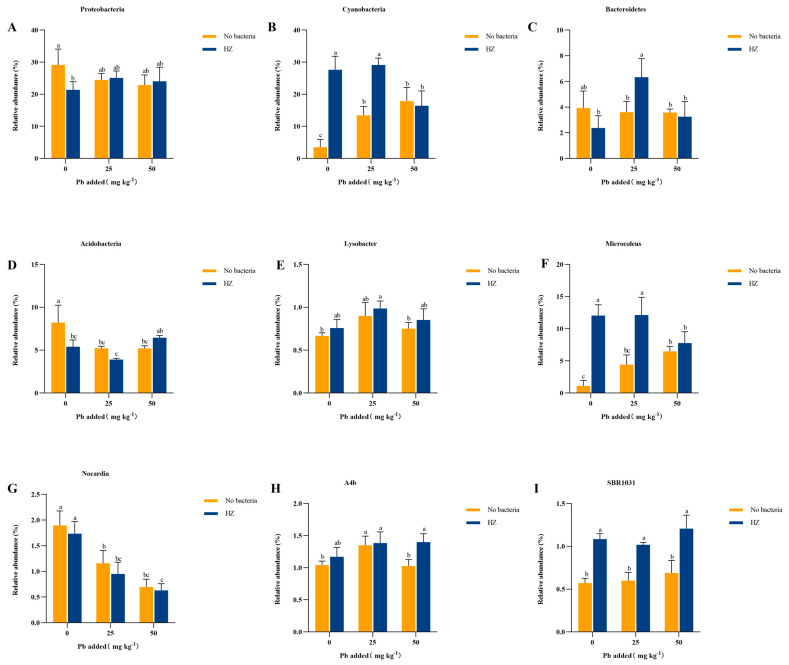
The influence of HZ on the relative abundance of some phyla in rhizosphere soil bacteria (**A**–**D**), and the influence of HZ on the relative abundance of some rhizosphere soil bacteria genera (**E**–**I**). Each bar represents the mean ± SD (n = 4). The same letter indicates no significant differences between the groups (*p* > 0.05).

Figure 7 illustrates the thermogram depicting the species composition of the top 30 phyla in the inter-root soil of pakchoi under varying Pb concentrations (Figure 7). In the absence of applied strains, the Pb-contaminated soil compared to the uncontaminated soil, *Latescibacteria*, *Nitrospirae*, *Patescibacteria*, *Verrucomicrobia*, *BRC1*, *Acidobacteria*, *Firmicutes*, *Rokubacteria*, *WPS-2*, *Planctomycetes*, *Armatimonadetes*, *Proteobacteria*, and *Gemmatimonadetes* decreased in abundance, and the application of strain HZ increased the abundance *Proteobacteria*, *Cyanobacteria*, *Bacteroidetes*, and *Acidobacteria* (Figure 8A–D). The changes in the inter-root soil bacterial flora across different treatments at the genus level are shown in the figure (Figure 7 and Figure 8E–I). Dominant genera of soil bacteria in the inter-roots of Chinese cabbage analyzed at the genus level primarily included *Microcoleus*, *Pseudomonas*, *Rhizobium*, *Lysobacter*, *Mycobacterium*, *Proteobacteria*, *Mycobacterium*, *Aeromicrobium*, and *Bacillus*. In addition, HZ can significantly improve *Lysobacter*, *Microcoleus*, *A4b*, and *SBR1031* relative abundance. The relative abundance of *Nocardia* decreased with the increase in Pb concentration, and the relative abundance of *Nocardia* was significantly lower than that of the control group when strain HZ was applied.

## 4. Discussion

EPSs display a robust affinity for polyvalent cations [28,33] and effectively adsorb heavy metals, capable of forming complexes with metal ions [17,34]. EPSs have demonstrated significant potential for heavy metal adsorption. Nonetheless, limited research has been conducted on the direct application of EPS-generating marine bacteria for soil heavy metal pollution remediation through EPS production. In this study, we investigated the remediation capabilities of EPS-producing bacteria, specifically strain HZ, in soil contaminated with heavy metals. This study investigated its ability to immobilize Pb in soil and examined its effects on growth quality, biomass, heavy metal content, physicochemical properties of inter-root soil, heavy metal content in the inter-root soil, and microbial community in the inter-root soil of pakchoi. The results revealed that strain HZ produced EPSs, which significantly reduced Pb content in the soil filtrate, mitigated Pb stress on Chinese cabbage, increased pakchoi biomass, and increased the content of vitamin C and soluble proteins in edible tissues, thereby improving pakchoi quality. Strain HZ also decreased Pb content in roots and edible tissues, as well as effective state Pb content in inter-root soil, increased soil pH, enhanced soil enzyme activity, and increased soil nitrogen content. Furthermore, alterations were observed in the variety of soil microbiomes and the prevalence of predominant bacterial phyla in the cabbage rhizosphere.

Nitrogen is vital for life as it serves as a crucial building block in plants [35,36], contributing to the formation of proteins, vitamins, hormones, and other essential components. The primary forms of nitrogen uptake by plants are ammonium nitrogen and nitrate nitrogen, which are readily assimilated. The uptake of NO_3_^−^ by plants is accompanied by the uptake of protons (H^+^), which leads to an increase in inter-root pH [37]. In addition, HZ increases urease activity in soil, and urea-producing bacteria can hydrolyze urea into ammonium salts and carbonate ions, thereby increasing soil carbonate concentration and pH. This is also consistent with the experimental results showing that HZ increases the soil filtrate and soil pH value (Figure 1E and Figure 4C). Inoculation with strain HZ improved the biomass and quality of pakchoi, likely owing to its ability to effectively increase ammonium and nitrate nitrogen content in the soil. Mei Peipei et al. found that inoculation with NM353 significantly increased aboveground nitrogen accumulation in broad beans [38]. Li Jiahuan et al. found that rhizobial inoculation increased the number of alfalfa nodules, aboveground biomass, and protein content, and decreased the underground biomass. Zhu et al. found that NRT1.1-mediated NO_3_^−^ uptake was specifically stimulated under Pb stress, increasing the inter-root pH and reducing plant Pb uptake [39]. These studies collectively validate that functional bacteria can effectively mitigate heavy metal damage and enhance plant growth, consistent with the results of this study.

Soil enzyme activity can reflect the intensity of biochemical reaction processes and the efficiency of soil nutrient transformation in a particular soil ecosystem, and indirectly reflect the biological activity of the soil. This serves as a crucial marker for assessing the behavior and vitality of soil ecosystems exposed to heavy metal contamination. Alkaline phosphatase originating primarily from soil microbes, catalyzes the mineralization of organic phosphorus in soil, aiding plant absorption and use [40]. Urease activity is closely associated with soil nitrogen transformation processes, while dehydrogenases catalyze the oxidation-reduction reactions of sugars, organic acids, and amino acids, crucial for soil respiration. In this study, we found that the activities of these enzymes decreased with increasing Pb stress, but significantly increased in the inter-root soil inoculated with HZ (Figure 4). These results showed that colonization by foreign bacteria could improve soil chemical reactions and plant growth [41,42,43]. Similarly, Wang et al. found that increased enzyme activities (soil alkaline phosphatase, urease, and dehydrogenase) and plant biomass in Cd-contaminated inter-root soils inoculated with the γ-PGA-producing bacteria [44]. Han et al. demonstrated that strains N3 and H12 immobilized Cd and Pb via extracellular adsorption and bioprecipitation, improving soil pH, reducing Cd and Pb levels in Brassica napus leaves (edible tissue), and increasing Brassica napus biomass [45].

Microorganisms in the soil between plant roots play a key role in regulating the carbon and nitrogen cycles for plant growth [12,46,47,48]. Plant growth depends heavily on these microorganisms. Numerous studies have indicated a notable reduction in plant inter-root microbial biomass when subjected to environmental stressors [47,49]. These microorganisms exist within a complex structural framework influenced by numerous elements such as soil chemistry, plant genetics, physical characteristics, and disturbances in the surrounding non-living environment [50,51]. The involvement of inter-root microbial community structure in the microbial immobilization of heavy metals remains to be understood because of unknown mechanisms. Researchers have found that Cd hyperaccumulating (enriched) plants growing in different soils have a unique set of core microbial communities in the inter-root, while different Cd hyperaccumulating (enriched) plants share a set of core microbes [52]. Soil bacteria can be modified by altering bacterial communities. Soil bacteria can adjust to prolonged heavy metal contamination by altering the taxonomic composition and structure of bacterial communities while preserving the functional diversity and ecological significance of the microbiota [53,54,55,56]. In this study, inoculation with HZ did not modify the species of the predominant phyla, but affected their respective abundance levels. The bacterial α-diversity indices in both Pb 0 mg kg^−1^ and Pb 25 mg kg^−1^ inter-root soils were lower than those of the control group (Table 2), suggesting that the exogenous strain HZ altered the diversity of the soil inter-root microbial community. This also suggests that the composition of inter-root soil microorganisms was not only affected by Pb stress but also by the modification of strain HZ. In our study, we observed that Pb contamination decreased the prevalence of *Proteobacteria*, *Chloroflexi*, *Acidobacteria*, *Bacteroidetes*, and *Firmicutes* in inter-root soil. This is similar to the results of a previous study, in which heavy metal-contaminated soil decreased the abundance of *Bacteroidetes*, *Proteobacteria*, and *Chloroflexi* [57,58]. Soil contaminated with heavy metals showed decreased abundances of *Proteobacteria*, *Chloroflexi*, and *Bacteroidetes*. *Actinobacteria*, *Chloroflexi*, *Aspergillus*, *Cyanobacteria*, and *Acidobacteria* were the dominant phyla in heavy metal-contaminated soil. In the inter-root soil inoculated with HZ, we found a significant increase in the abundance of *Proteobacteria*, *Chloroflexi*, and *Cyanobacteria* in the soil community, which may also be due to their higher tolerance to heavy metals [56,59]. Microorganisms in the *Cyanobacteria* increase the total nitrogen content of soil through nitrogen fixation. *Actinomycetes* can decompose and utilize organic matter, thereby contributing to the soil carbon cycle [60]. This significantly contributes to the carbon cycle in the soil [61]. *Proteobacteria*, *Acidobacteria*, and *Actinobacteria* are essential for soil nutrient cycling and heavy-metal remediation. These bacterial communities are intricately linked to soil organic matter, nitrogen availability, phosphorus availability, and heavy metals. Additionally, we observed that Pb contamination affected the bacterial community at the genus level. After inoculation with HZ, the relative abundances of *Microcoleus*, *Pseudomonas*, *Rhizobium*, *Lysobacter*, *Mycobacterium*, *Aeromicrobium*, and *Bacillus* were relatively high. Members of these genera are usually metal-tolerant species that promote crop growth [62]. The genera *Aeromicrobium*, *Bacillus*, and *Bacillus* are relatively abundant. It was hypothesized that these phyla and genera play important roles in blocking Pb uptake by pakchoi. These results suggest that it is feasible to utilize the extracellular polysaccharide-producing bacterium HZ for soil remediation of heavy metal contamination.

## 5. Conclusions

Research into remediation technology for heavy-metal-contaminated soil is currently in a developmental stage and remains a key research topic. In this study, we found that the extracellular polysaccharide-producing bacterium HZ effectively increased soil pH and immobilized Pb in heavy metal-contaminated soil. Furthermore, HZ enhanced the biomass, Vc, and soluble protein content of pakchoi, thereby improving its overall quality. It is hypothesized that strain HZ may reduce the effective Pb content in edible tissues, roots, and inter-root soil by regulating the structure of microbial communities in inter-root soil, increasing soil pH, nitrogen content, and soil enzyme activity, and changing the abundance of dominant phyla. The findings of this study underscore the significant potential of HZ in mitigating Pb absorption in pakchoi and remediating soils contaminated with heavy metals.

## Figures and Tables

**Table 1:** Sequences of 16SrRNA high-throughput sequencing of inter-root soil bacterial samples.

Treatment	Raw Sequences	Qualified (No.)
Pb0CK_1	139073	82,793
Pb0CK_2	151032	80,818
Pb0CK_3	126493	69,484
Pb0CK_4	129797	87,466
Pb0HZ_1	131850	77,391
Pb0HZ_2	130246	79,238
Pb0HZ_3	128867	68,490
Pb0HZ_4	148993	67,085
Pb25CK_1	136723	87,648
Pb25CK_2	129863	74,599
Pb25CK_3	126683	75,559
Pb25CK_4	146233	57,391
Pb25HZ_1	123545	69,729
Pb25HZ_2	117928	65,192
Pb25HZ_3	119091	63,747
Pb25HZ_4	153962	62,999
Pb50CK_1	133478	72,788
Pb50CK_2	126592	74,589
Pb50CK_3	122184	58,901
Pb50CK_4	148932	66,457
Pb50HZ_1	122266	74,712
Pb50HZ_2	136385	84,387
Pb50HZ_3	130485	78,091
Pb50HZ_4	140886	53,173

**Table 2:** Effect of strain HZ on the α-diversity index of inter-root soil bacterial communities of pakchoi at different Pb concentrations.

Pb Added (mg kg^−1^)	Chao1	Observed Species	Shannon	Simpson
0 *				
CK	7619 ± 724 a	6456 ± 489 a	11.1 ± 0.3 a	0.998 ± 0.0009 a
HZ	6399 ± 1088 a	5383 ± 839 a	10.1 ± 0.9 ab	0.9928 ± 0.0051 ab
25 *				
CK	6979 ± 590 a	5827 ± 638 a	10.7 ± 0.3 ab	0.9975 ± 0.0007 a
HZ	5586 ± 1539 a	4802 ± 1200 a	9.4 ± 1.1 b	0.9838 ± 0.0104 b
50 *				
CK	7088 ± 472 a	5903 ± 225 a	10.6 ± 0.2 ab	0.9961 ± 0.002 ab
HZ	7144 ± 289 a	6078 ± 269 a	10.8 ± 0.4 ab	0.9965 ± 0.0018 a

* Data followed by the same letter (a–b) within the same row are not significantly different by Tukey’s test (*p* > 0.05).

## Data Availability

The data presented in this study are available on request from the corresponding author. The data are not publicly available as these data also form part of an ongoing study.
